# A Nursing Manual for Nurses and Nursing Orderlies

**Published:** 1915-12

**Authors:** 


**A Nursing Manual for Nurses and Nursing Orderlies.** By DUNCAN C. L. FITZWILLIAMS, M.D., Ch.M., F.R.C.S. Pp. vii. 466. London: Oxford Medical Publications. 1914. Price 6s. net.

Of the making of books specially written for nurses there seems no end, and the volume under review is a typical example of its class. The captious critic searches in vain for inaccuracies or omissions, while the style is eminently readable. The arrangement may be described as regional, each system being treated by itself, and a description of the diseases of the system being preceded by an account of its anatomy and physiology. There is much to be said for this arrangement, even if, on casual inspection, it seems to involve rather a mixture of subjects. To give an instance of what is meant, a description of a bed-pan immediately precedes, on the same page, an account of the pathology of infantile paralysis.

To sum up, it may be said that the author has produced a volume which contains an enormous amount of information, written in a style which should make it readily assimilable by the average nurse.

When she knows all the book contains, and how to apply her knowledge, her theoretical training should leave little to be desired. A complete and accurate index is a valuable feature.

